# Single-cell transcriptomics of melanoma sentinel lymph nodes identifies immune cell signatures associated with metastasis

**DOI:** 10.1172/jci.insight.183080

**Published:** 2025-03-06

**Authors:** Eric Engelbrecht, Bryce F. Stamp, Lewis Chew, Omar Sadi Sarkar, Phillip Harter, Sabine J. Waigel, Eric C. Rouchka, Julia Chariker, Andrei Smolenkov, Jason Chesney, Kelly McMasters, Corey T. Watson, Kavitha Yaddanapudi

**Affiliations:** 1 Department of Biochemistry and Molecular Genetics;; 2 Immuno-Oncology Group, UofL-Health Brown Cancer Center;; 3 Department of Microbiology/Immunology;; 4 Department of Medicine;; 5 Department of Computer Science and Engineering;; 6 Department of Neuroscience, KBRIN Bioinformatics Core;; 7 UofL-Health Brown Cancer Center;; 8 Division of Surgical Oncology, Department of Surgery; and; 9 Division of Immunotherapy, Department of Surgery, University of Louisville, Louisville, Kentucky, USA.

**Keywords:** Immunology, Oncology, Melanoma

## Abstract

The sentinel lymph node (SLN) is the first lymph node encountered by a metastatic cancer cell and serves as a predictor of poor prognosis, as cases with clinically occult SLN metastases are classified as stage III with elevated rates of recurrence and diminished overall survival. However, the dynamics of immune infiltrates in SLNs remain poorly characterized. Here, using an unbiased cellular indexing of transcriptomes and epitopes by sequencing technique, we profiled 97,777 cells from SLN tissues obtained from patients with stages I/II and III cutaneous melanoma. We described the transcriptional programs of a multitude of T, B, and myeloid cell subtypes in SLNs. Based on the proportions of cell types, we determined that SLN subtypes stratified along a naive → activated axis; patients with a “high activated” signature score appeared to be undergoing a robust melanoma antigen–driven adaptive immune response and, thus, could be responsive to immunotherapy. Additionally, we identified transcriptomic signatures of SLN-infiltrating dendritic cell subsets that compromise antitumor immune responses. Our analyses provide valuable insights into tumor-driven immune changes in the SLN tissue, offering a powerful tool for the informed design of immune therapies for patients with high-risk melanoma.

## Abstract 2

Here, we used high-dimensional CITE-seq technique to map the immune landscape of sentinel lymph<br> nodes, aiding the design of novel immunotherapies for early-stage melanoma patients.

## Introduction

Immune checkpoint blockade (ICB) agents have revolutionized the landscape of cancer immunotherapeutics and can provide durable responses in patients with advanced cancer (1). Nevertheless, there is an outstanding need for improved treatment regimens, as many patients do not achieve long-term clinical benefits (1). To this end, it is critical to develop therapeutic approaches that not only trigger a strong immune response but also establish durable and functional memory T and B cell populations capable of safeguarding against disease recurrence or relapse. Tumor-draining lymph nodes (LNs) are probably key sources of adaptive antitumor responses (2–5); meanwhile, they also serve as conduits for metastasis and are often resected early in the course of disease.

Identification of melanoma cells in the most proximal tumor-draining LN, termed the sentinel LN (SLN), distinguishes stage III (SLN^+^) from stage I/II (SLN^–^) disease. SLN resection near the time of diagnosis removes not only metastatic melanoma cells but also diverse memory/effector immune cell populations that, if activated, may promote antimelanoma systemic immunity (6). Despite the importance of SLNs in melanoma management, we lack an understanding of the immune landscape of SLNs in stage I/II melanoma and how this tissue may be altered after cancer cell invasion.

Melanoma cases with a single microscopically positive SLN are classified as stage III despite the fact that there is substantial variation in disease recurrence in these cases (7, 8). Furthermore, patients with ulcerated cutaneous melanomas have a greater risk of SLN metastasis and recurrence (8). As such, a gap exists in our understanding of whether or how we can reshape the immune landscape of melanoma SLNs and promote systemic tumor-specific immunity in patients with early-stage cancer who are at high risk for disease recurrence.

Studies to date have shown that melanoma development and disease spread signal changes in the SLN microenvironment that are associated with regional immune tolerance (9). These alterations include both decreases in the number and activity of dendritic cells (DCs) and increases in infiltration of T regulatory cells (Tregs) that work in tandem to suppress effective T cell–dependent antitumor immune responses (10–12). In our recent work (5), using a combination of mass cytometry, flow cytometry, and TCR-sequencing techniques, we compared the immune landscape of melanoma-negative (stage I) with melanoma-bearing (stage III) SLNs. Our study identified antimelanoma T cell functional signatures (characterized by clonal expansion of effector-memory αβ T cells) as well as an immunotolerant microenvironment (characterized by reduced NK cell cytolytic function and enrichment of T cell subsets with poor cytolytic potential) in the melanoma-bearing SLNs (5). However, this approach lacked the ability to simultaneously capture both gene and protein information. In this study, we have addressed this limitation by utilizing the cellular indexing of transcriptomes and epitopes by sequencing (CITE-Seq) technique (13) to determine the transcriptional states of diverse immune cell populations within the tumor-free and tumor-bearing SLN microenvironments. CITE-Seq integrates single-cell RNA sequencing with the multiplexed measurement of surface proteins at the single-cell level.

Our analysis provides a relatively unbiased and comprehensive map of both phenotypic and transcriptional programs of tissue-resident and migratory T and B cell subsets in SLNs. In patients with high-risk stage III melanoma, we found T cell phenotypes shifted from an activated to a more terminally differentiated exhaustive phenotype. This immune pattern may be responsive to administration of ICB therapy and suggests that a neoadjuvant immunotherapy strategy before SLN biopsy may provide clinical benefit.

## Results

### Profiling immune infiltrates in human melanoma SLN tissues using CITE-Seq.

**Figure 1:**
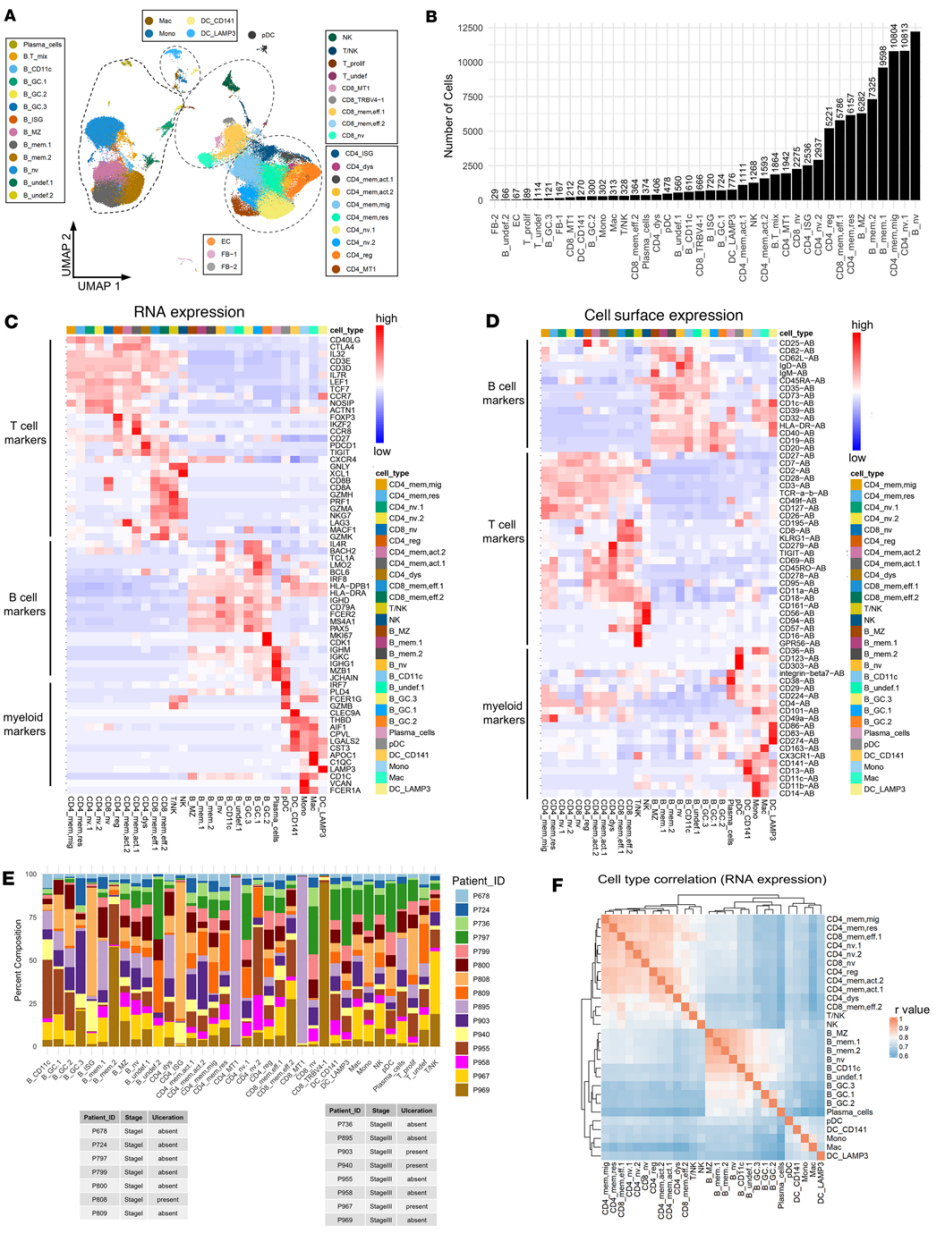
Deciphering the diversity of immune cells in melanoma SLN tissues using CITE-Seq. (**A**) Uniform manifold approximation and projection (UMAP) plot of cells from SLNs, colored by cluster. (**B**) Bar plot of cell number in each cluster. (**C**) RNA and cell surface (**D**) expression heatmaps of selected cluster markers (Supplemental Tables 2 and 3). (**E**) Stacked bar indicating the relative contribution of each patient (color) to each cluster. (**F**) Heatmap of cell type correlations (*r* values) based on RNA expression for 5,000 genes. Mac, macrophage; Mono, monocyte; DC, dendritic cell; pDC, plasmacytoid DC; GC, germinal center; ISG, IFN-stimulated gene; MZ, marginal zone; MT, metallothionein; EC, endothelial cell; FB, fibroblast.

To probe the transcriptional states of immune cells in the SLN microenvironment, we profiled cells from SLN samples resected from melanoma patients diagnosed with pathological stage I/II (henceforth referred to as stage I, melanoma-negative LNs, or MEL^–^ SLNs) and stage III disease (melanoma-positive LNs, or MEL^+^ SLNs) (Scheme I, Supplemental Methods; supplemental material available online with this article; https://doi.org/10.1172/jci.insight.183080DS1). CITE-Seq libraries were prepared individually for each donor sample (7 MEL^–^ SLNs and 8 MEL^+^ SLNs; 1 LN sample from each patient). After quality control filtering (see Methods), a range of 3,378 to 10,327 cells were analyzed from each patient sample (Supplemental Table 1) for a total of 97,777 cells. In Figure 1, A–F, we pooled all 97,777 cells from 15 patient samples (7 MEL^–^ SLNs and 8 MEL^+^ SLNs; 1 LN sample from each patient) and performed clustering using Seurat as described in the Methods. No training set was utilized. Available details related to each donor, including SLN status (disease stage), age at the time of sample collection, sex, presence/absence of ulceration in the SLN, and Breslow thickness, are included in Supplemental Table 1. Also included in Supplemental Table 1 is the number of cells from each cluster from each patient sample. Unsupervised clustering based on levels of RNA expression revealed a predominance of B and T lymphocytes in addition to smaller clusters of monocytes, macrophages, and DCs (Figure 1, A and B). Clusters were distinguished by expression of known cell type markers, including those for CD4^+^ T cells (*CD40LG*, CD3, CD4), CD8^+^ T cells (*CD8A*, CD3, CD8), NK cells (*XCL1*, CD56, CD16), B cells (*MS4A1*, CD19, CD20), plasma cells (*MZB1*, *JCHAIN*, CD38), pDCs (*IRF7*, *PLD4*, *GZMB*, CD123, CD303), CCR7^+^/LAMP3^+^ DCs (*CCR7*, *LAMP3*, CD1C, PD-L1/CD274), CD141^+^ DCs (*CLEC9A*, *THBD*/CD141), macrophages (*C1QC*, *APOC1*, CD163), and monocytes (*VCAN*, *FCER1A*, CD11b) (Figure 1, C and D, and Supplemental Tables 2 and 3). A small population of proliferating T cells (T_prolif) was distinguished by expression of cell cycle–associated genes (*MKI67*, *STMN1*) (Supplemental Table 2). A cluster expressing markers of both B and T cells (B.T_mix) (Supplemental Table 2) was unresolvable and disregarded from downstream analyses. Additionally, endothelial cells (*PECAM1^+^*, *PLVAP^+^*) and fibroblasts (*DCN^+^*, *COL1A1^+^*), which together composed 0.27% of cells (Figure 1B and Supplemental Table 2), were also disregarded from downstream analyses.

To define lymphocyte populations, we separated B cells, CD4^+^ T cells, and CD8^+^ T cells into independent groups and performed differential expression analysis to identify transcripts enriched in each cluster (Supplemental Tables 4–7). This analysis identified gene expression differences among 10 CD4^+^ T cell clusters, 5 CD8^+^ T cell clusters, and 11 B cell clusters. Most clusters were composed of cells from each patient (Figure 1E; from 15 patients, 1 LN sample from each patient; colors represent patient samples, and *x* axis categories indicate cell types). However, clusters CD4_ISG, B_ISG, CD4_MT1, CD8_MT1, and CD8_TRBV4-1 were predominantly derived from 1 or 2 patients (Figure 1E and Supplemental Table 1) and were characterized by high expression of ISGs, MT-encoding genes, or TRBV4-1 (Supplemental Table 2); these clusters were not carried forward for downstream analysis. Gene expression correlation of cell types revealed separation of naive from memory/effector T cell populations (*n* = 5,000 genes; from 15 patients, 1 LN sample from each patient) (Figure 1F). Spearman’s *r* values (Figure 1F) were computed using the “clustify” command of the clustifyr package with default parameter, as described in the Methods.

### Features of cell activation and exhaustion in CD8^+^ T cell and NK populations within SLNs.

**Figure 2:**
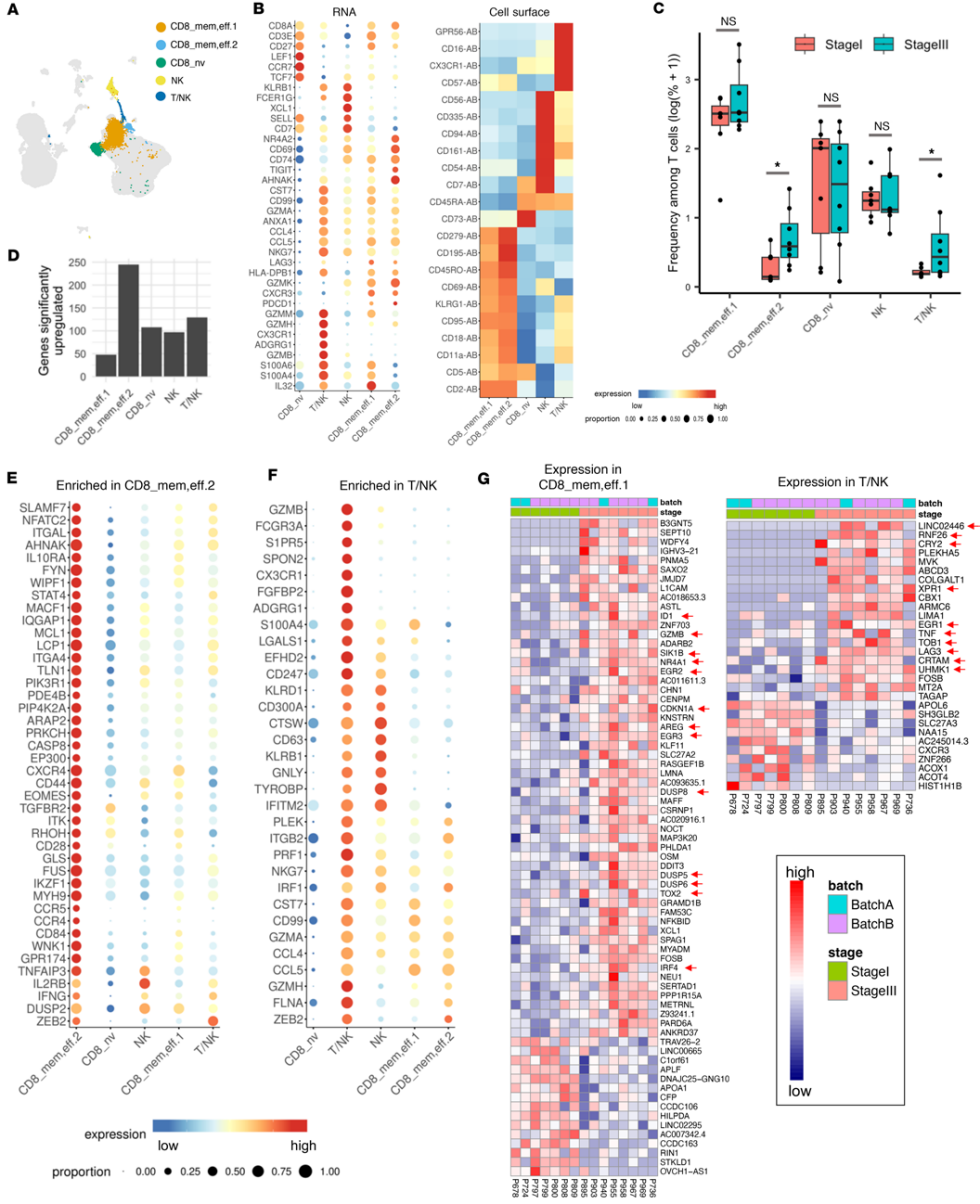
Melanoma disease stage associates with transcriptional state of CD8^+^ effector T cells in the SLN tissues. (**A**) UMAP plot with CD8^+^ and NK cells highlighted. (**B**) Dot plots of RNA (left) and heatmap of cell surface (right) expression of selected genes upregulated in CD8^+^ T cell and NK clusters. (**C**) Box plot (median with interquartile range) showing the frequency of CD8^+^ T cell and NK clusters among all T cells. *P < 0.05. (**D**) Bar plot of the number of genes upregulated (log_2_ fold-change > 0.4 and Padj < 5.0 × 10^–7^) in indicated clusters. (**E** and **F**) RNA expression dot plots of selected genes markedly upregulated in CD8_mem,eff.2 (**E**) and T/NK (**F**) cells. (**G**) Heatmap showing expression of all transcripts that are differentially expressed (*P* value < 0.01 and |log_2_ fold-change| > 0.8) between stage I and stage III SLN samples in CD8_mem,eff.1 (**G**; left) and T/NK (**G**; right) cells. Red arrows in **G** indicate genes that are associated with CD8^+^ T cell anergy, exhaustion, suppression, T cell immune regulation, and lymph node retention. Two-group comparisons were done by 2-tailed Student’s *t* tests.

Next, we analyzed the different T lineages present in SLNs across all patients. Figure 2A shows the UMAP clustering plot colored using CD8, NK, and T/NK cell channels (from all 97,777 cells from 15 donors; 7 MEL^–^ SLNs and 8 MEL^+^ SLNs; 1 LN sample from each patient). In Figure 2B, average expression among all cells belonging to each T cell type (*x* axis categories) is plotted. Naive CD8^+^ T cells were marked by high expression of *LEF1* and *CCR7* in addition to the cell surface protein CD73 (Figure 2, A and B), an ecto-5′-nucleotidase centrally involved in generating adenosine, a potent immunosuppressive molecule in the tumor microenvironment (14). We identified 2 distinct effector memory CD8^+^ T cell populations, CD8_EM1 (CD8_mem,eff.1; 5,786 cells) and CD8_EM2 (CD8_mem,eff.2; 364 cells) (Figure 2, A and B). While CD8_EM1 cells were identified at similar frequencies in stage I MEL^–^ and stage III MEL^+^ SLNs, the CD8_EM2 cluster was enriched in MEL^+^ SLN biopsies (Figure 2C).

Differential expression analysis among T and NK cell populations revealed that the CD8_EM2 cluster harbored the greatest number of markedly upregulated genes (Figure 2D and Supplemental Table 4), indicating that CD8_EM2 cells express a distinct set of genes as compared with the remainder of CD8^+^ T cells. Both CD8_EM1 and CD8_EM2 clusters expressed genes associated with CD8^+^ T cell effector function (*CXCR3*, *GZMK*, *GZMA*, *CCL4*, *SLAMF7*) and activation (*CD69*, *AHNAK*, *CD44*, *EOMES*, *TLN1*, *PRKCH*, *IFNG*, *IL2RB*, *STAT4*); however, CD8_EM2 cluster showed higher expression of these genes that are involved in effector function and activation (Figure 2, B and E). CD8_EM2 cells also expressed high levels of genes associated with T cell migration (*CCR4*, *ITGA4*, *CXCR4*, *WIPF1*, *MYH9*, *MACF1*, *CD44*, *DUSP2*, *RHOH*, *LCP1*), survival (*MCL1*), transcription activation (*EP300*), and suppression (*PDCD1*/PD-1, *CD84*, *NR4A2*, *IL10RA*, *TNFAIP3*, *GPR174*, *IQGAP1*) (Figure 2, B and E). When compared with the CD8_EM1 cluster, CD8_EM2 cells displayed a more terminally differentiated/exhausted phenotype with higher expression of coinhibitory markers *LAG3*, *TIGIT*, and *IKZF1* (Figure 2, B and E), whereas CD8_EM1 cells were distinguished by high expression of *IL32* (Figure 2B). While the precise function of IL-32 in these cells remains uncertain, a recent study (15) reported that IL-32 treatment reduced tumor growth and rendered ICB-resistant murine melanoma tumors responsive to anti–programmed cell death 1 (anti–PD-1) therapy. Furthermore, a higher baseline expression of the IL-32 gene was associated with a positive response to ICB therapy in patients with melanoma (15).

Cell surface PD-1 (CD279) was detected on both CD8_EM1 and CD8_EM2 cells (Figure 2B), consistent with our previous study of melanoma SLNs (5). Both of these clusters also expressed *CXCR3* (Figure 2B), a receptor for IFN-stimulated chemokines CXCL9 and CXCL10 expressed on activated T cells (16–18). Since the CXCR3 chemokine system was reported to be indispensable for the anti–PD-1–mediated response of the CD8^+^ T cell population residing within melanoma tumors (19), we analyzed the expression of CXCR3 on CD8^+^PD-1^+^ T cells in the SLN tissue by flow cytometry. Based on the expression of CXCR3 and PD-1, 3 subsets of CD8^+^ T cells were identified in the SLN tissues: CXCR3^+^PD-1^–^, CXCR3^+^PD-1^lo^, and CXCR3^+^PD-1^intermediate^ (Supplemental Figure 1, A and B). Furthermore, our data revealed an association between PD-1 expression and expression of additional immune checkpoint/T cell activation–related molecules (TIGIT, CD28, LAG3) (Supplemental Figure 1, C and D). These data demonstrate that effector-memory CD8^+^ T cells present in the SLN tissue display features of both memory/effector function and exhaustion, suggesting that these cells could be reactivated by inhibition of immunosuppressive signaling pathways.

Given that the expression of circulating CXCR3 ligands CXCL9 and CXCL10 is a strong predictor of clinical responses to ICB therapy in patients with late-stage melanoma (19), we investigated if circulating levels of CXCL9 and CXCL10 were altered in stage I versus stage III melanoma. Using multiplex Bio-Plex technology, we analyzed the expression of CXCL9 and CXCL10 chemokines in serum samples from healthy donors and patients with stage I and stage III melanoma. Compared with healthy donors and stage I patients, the stage III group had elevated serum levels of CXCL10 (Supplemental Figure 1E), a prototypical ISG product (18). These data suggest that circulating ISG-CXCL10 chemokine can be an early biomarker for response to immunotherapy. None of the other 38 soluble cytokines and chemokines analyzed (Supplemental Table 3) were markedly altered in the sera from stage I versus stage III patients (Supplemental Table 9).

### CD8^+^ effector-memory T cells in melanoma-bearing SLNs are in an early dysfunctional state and exhibit cytolytic features.

As the CD8_EM1 cluster was the largest CD8^+^ T cell population present in the SLNs, we sought to determine how the phenotype of these cells is altered in the context of stage III versus stage I melanoma. Differential expression analysis (see Methods) revealed that the majority of differentially expressed genes were upregulated in stage III MEL^+^ SLN samples. Genes upregulated in CD8_EM1 cells in MEL^+^ SLNs were associated with T cell anergy (*IRF4*, *DUSP5*, *DUSP6*, *DUSP8*) (20, 21), exhaustion (*TOX2*, *EGR2*, *EGR3*, *NR4A1*) (22–27), and suppression (*ID1*) (28) (Figure 2G). These data indicate that CD8_EM1 cells exhibit features of a dysfunctional phenotype in MEL^+^ SLNs from stage III cases. We similarly determined genes that were differentially expressed between MEL^–^ and MEL^+^ SLN samples specifically within the T/NK cell populations, and our data revealed that genes associated with T cell immune regulation (*LAG3*) and LN retention (*CRTAM*) (29) were upregulated in T/NK cells present in MEL^+^ SLNs (Figure 2G). Taken together, these data suggest that MEL^+^ SLNs from stage III melanoma harbor CD8^+^ T cells that are primed to carry out effector functions but are skewed toward an early dysfunctional phenotype, which may contribute to the melanoma progression with growing metastases in the MEL^+^ SLNs but can be reinvigorated by immunotherapy.

MEL^+^ SLN biopsies from stage III patients were also enriched with a cluster of cells that showed a mixed CD8^+^ T cell and NK phenotype, exhibiting expression of NK markers (*KLRB1*, *KLRD1*, CD16, CD56) and effector CD8^+^ T cell markers (*GZMA*, *CCL4*, *PRF1*, CD11a) (Figure 2, B and F). Genes upregulated in T/NK cells that drove their identity include *GZMB*, *GZMH*, *S1PR5*, *SPON2*, *CX3CR1*, *FGFBP2*, and the immune checkpoint *ADGRG1*/GPR56 (30–32), expressed at the RNA level (Figure 2F). T/NK cells were also notable for high expression of cell surface CD57 (Figure 2B), which is consistent with our previous study of melanoma SLNs (5). Prior research has revealed that senescent and terminally differentiated effector cells, which demonstrate reduced in vivo antitumor activity, exhibit elevated levels of the CD57 marker (33, 34). Additionally, the transcription factor *ZEB2* was specifically expressed in T/NK and CD8_EM2 cells (Figure 2F), consistent with a role for ZEB2 in driving CD8^+^ T cell activation and differentiation toward an effector phenotype (35, 36). These data demonstrate that a cytotoxic GPR56^+^*CX3CR1*^+^*GZMB/H*^+^ T/NK cell population is enriched in MEL^+^ SLN biopsies and harbors features of cytotoxic potential.

### Melanoma SLNs harbor few CD3^–^ NK cells.

A CD3^–^ NK cell cluster in the SLNs was characterized by expression of cell surface markers CD56 and CD335 (NKp46, NCR1), as well as CD161 (Figure 2B). CD161 has been shown to inhibit cytotoxic capability while increasing the cytokine responses of NK cells (37–40). Other markers expressed on NK cells included CD94 (KLRD1), CD54, and CD7 (Figure 2B). Additional transcripts enriched in NK cells included *FCER1G* that encodes Fc fragment of IgE (receptor Ig), *KLRB1*, *SELL* (CD62L), and *CD7* (Figure 2B). The transcript encoding the chemokine XCL1 was substantially higher in NK cells. XCL1 binds to the XCR1 chemokine receptor and has been demonstrated to play a critical role in recruiting crosspresenting DCs to tumor tissues (41).

### CD4^+^ T cells in melanoma-bearing SLNs exhibit distinct features of activation, exhaustion, and cytolytic potential.

**Figure 3:**
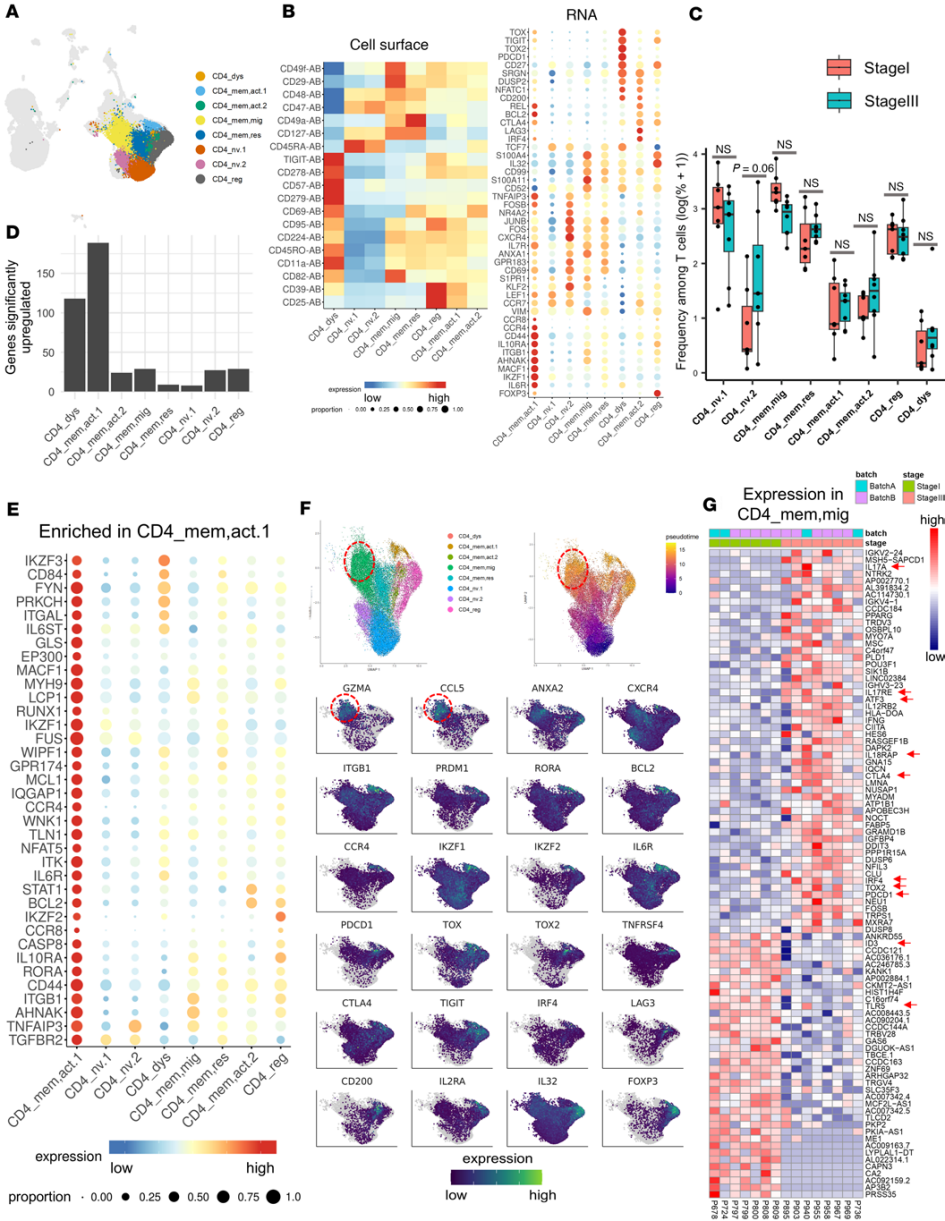
A dysfunctional CD4^+^ T cell cluster with features of migratory and cytotoxic activity is enriched in melanoma-bearing SLNs. (**A**) UMAP plot with CD4^+^ cells highlighted. (**B**) Heatmap of cell surface (left) and dot plot of RNA (right) expression for selected genes upregulated in CD4^+^ T cell clusters. (**C**) Box plot (median with interquartile range) showing the frequency of CD4^+^ T cell clusters among all T cells. (**D**) Bar plot of the number of genes upregulated (log_2_ fold-change > 0.4 and Padj < 5.0 × 10^–7^) in indicated clusters. (**E**) RNA expression dot plot of selected genes markedly upregulated in CD4_mem,act.1 cells. (**F**) Slingshot analysis of CD4^+^ T cell clusters (top) and RNA expression of indicated genes (bottom). Dashed ovals indicate a subpopulation of migratory/memory CD4^+^ T cells that express high levels of the cytolytic genes CCL5 and GZMA. (**G**) Heatmap showing expression of all transcripts that are differentially expressed (*P* value < 0.01 and |log_2_ fold-change| > 0.8) between stage I and stage III SLN samples in CD4_mem,mig cells. Red arrows in **G** indicate genes that are associated with CD4^+^ Th1 and Th17 differentiation and activation/exhaustion. Two-group comparisons done by 2-tailed Student’s *t* tests.

Among CD4^+^ T cell clusters (Figure 3A), memory T cells were distinguished by high cell surface expression of CD45RO, CD69, CD95, CD224, and CD49a (Figure 3B), whereas naive T cells showed high expression of *CCR7* (Figure 3B). Two naive CD4^+^ T cell populations were identified, CD4_nv1 and CD4_nv2, the latter expressing high levels of *KLF2*, *CXCR4*, *NR4A2*, *CD69*, *FOS*, *FOSB*, *JUNB*, *GPR183*, and *ANXA1*, among others (Figure 3B). Top markers of CD4_nv2 cells were also expressed in memory populations (Figure 3B), collectively suggesting that CD4_nv2 cells are in an activated state and that naive CD4^+^ T cells within SLNs can be organized along a gradient characterized by increasing expression of memory genes and transcription factors. A trend toward increased frequency of CD4_nv.2 cells was observed in MEL^+^ SLNs (Figure 3C).

We identified 5 CD4^+^ T cell clusters with memory features, which exist in a continuum of states: (a) CD4_mem,act1, (b) CD4_mem,act.2, (c) CD4_dys, (d) CD4_mem,mig, and (e) CD4_mem,res clusters. The most distinct among these clusters, designated as CD4_mem,act1 (memory, activated 1), showed upregulation of a larger number of genes when compared with their levels in the other CD4^+^ T cell clusters (Figure 3D). We noted that markers of CD4_mem,act1 cells were also upregulated in the CD8_EM2 cluster, including genes associated with activation (*AHNAK*, *CD44*, *TLN1*, *PRKCH*), T cell migration (*CCR4*, *MACF1*, *CD44*, *DUSP2*, *LCP1*), survival (*MCL1*), suppression (*CD84*, *IL10RA*, *TNFAIP3*, *GPR174*, *IQGAP1*), and transcription activation (*EP300*) (Figure 2E and Figure 3E), indicating shared features between these CD4^+^ and CD8^+^ memory T cell subtypes. In addition, CD4_mem,act1 cells expressed *CCR4* and *CCR8* (Figure 3E), which are markers of skin-homing and skin-resident memory T cells, respectively (42, 43).

A cluster of CD4^+^ T cells were distinguished by high levels of *IRF4* (activation/differentiation), *LAG3*, and *CTLA4* (inhibitory/activation); therefore, these cells were designated as a second cluster of CD4 memory/activated cells (CD4_mem,act.2) (Figure 3B). On UMAP, these cells clustered closer to a relatively smaller cluster (*n* = 406 cells) of dysfunctional CD4^+^ T cells (CD4_dys) (Figure 3A), marked by high expression of *PDCD1*/PD-1, *TOX*, *TOX2*, and *TIGIT* (Figure 3B).

A fourth memory CD4^+^ T population was termed migratory (CD4_mem,mig) because of high expression of genes associated with T cell egress (*S1PR1*, *ITGB1*, CD62L, CD82, CD49f) and cell migration (*VIM*, *S100A4*, *S100A11*) (Figure 3B). This migration-associated CD4^+^ T population trended toward decreased frequency in MEL^+^ SLN biopsies (Figure 3C). For additional insight into CD4^+^ T cell heterogeneity, we performed pseudotime analysis (Monocle3) (44) rooted with naive CD4^+^ T cells (CD4_nv1) (Figure 3F). While markers of pseudotime generally recapitulated cluster markers (Figure 3F), we noted a subpopulation of migratory/memory CD4^+^ T cells (CD4_mem,mig) at a terminus of pseudotime that uniquely expressed high levels of the cytolytic genes *CCL5* and *GZMA* (Figure 3F). We sought to determine how the phenotype of CD4_mem,mig cells is altered in the context of stage III versus stage I melanoma (Figure 3G). CD4_mem,mig cells derived from MEL^+^ SLN biopsies upregulated genes associated with Th1 (*IFNG*, *IL18RAP*, *IL12RB2*, *ATF3*) (45) and Th17 (*IL17A*, *IL17RE*) differentiation, as well as activation/exhaustion (*TOX2*, *PDCD1*/PD-1, *CTLA4*, *IRF4*, *DUSP6*, *DUSP8*) (Figure 3G). Among genes expressed at higher levels in MEL^–^ SLN tissues were *ID3*, associated with a memory Th1 phenotype (46), and *TLR5*, agonists of which promote antitumor immunity (Figure 3G) (47).

Analysis of the CD4^+^ Treg cluster revealed a coregulated gene expression pattern that included transcription factors *FOXP3* and *IKZF2*, costimulatory receptor *CD27*, cytokine signaling receptors *IL-10RA* and *IL32*, and apoptosis regulator *BCL2*. Tregs displayed prototypical cell surface markers CD25 and CD95 (Figure 3, B and E). Tregs also expressed high levels of CD39 (Figure 3B), an ectonucleotidase that enzymatically converts ATP to AMP (48). Along with CD73, CD39 participates in the generation of immunosuppressive extracellular adenosine (49). Furthermore, some of the genes expressed in Tregs overlapped with those expressed in dysfunctional CD4^+^ T cells. These genes included coinhibitory and costimulatory receptors *TIGIT*, *TOX*, *CTLA4*, and *CD27* (Figure 3B) while immune checkpoint and regulatory genes *PDCD1* (PD-1), *TOX2*, *DUSP2*, *SRGN*, *NFATc*, and *CD200* were preferentially enriched in the dysfunctional cells (Figure 3B). *TCF7*, which encodes the transcription factor TCF-1, was markedly downregulated in the Treg cluster but enriched in dysfunctional CD4^+^ T cells (Figure 3B). These data indicate similarity between CD4^+^PD-1^+^TCF-1^+^ dysfunctional cells and previously described progenitor exhausted cells (50), which are characterized by the retention of polyfunctionality, long-term persistence, and ability to limit tumor growth as compared with terminally exhausted T cells. Recent studies have shown that progenitor exhausted T cells can respond to anti–PD-1 therapy, while the terminally exhausted T cells do not (50–53). Furthermore, in patients with melanoma, a higher proportion of progenitor exhausted cells correlates with an extended duration of response to ICB therapy (50). Consequently, strategies aimed at expanding the population of progenitor exhausted CD4^+^ T cells in the SLN tissues may prove crucial to increasing responsiveness to ICB therapy and controlling melanoma progression.

Taken together, these observations indicate that while the CD4^+^ T cell composition is largely similar between MEL^–^ and MEL^+^ SLN biopsies, there is evidence of increased activation, differentiation, and exhaustion phenotype in CD4^+^ T cells present in the MEL^+^ SLNs. In addition, tumor-specific cytolytic CD4^+^ T cells, which have been previously identified (54) in tumor-bearing hosts, may receive activation signals in melanoma-draining SLNs and can influence antitumor effector functions.

### B cell populations in melanoma SLNs.

**Figure 4:**
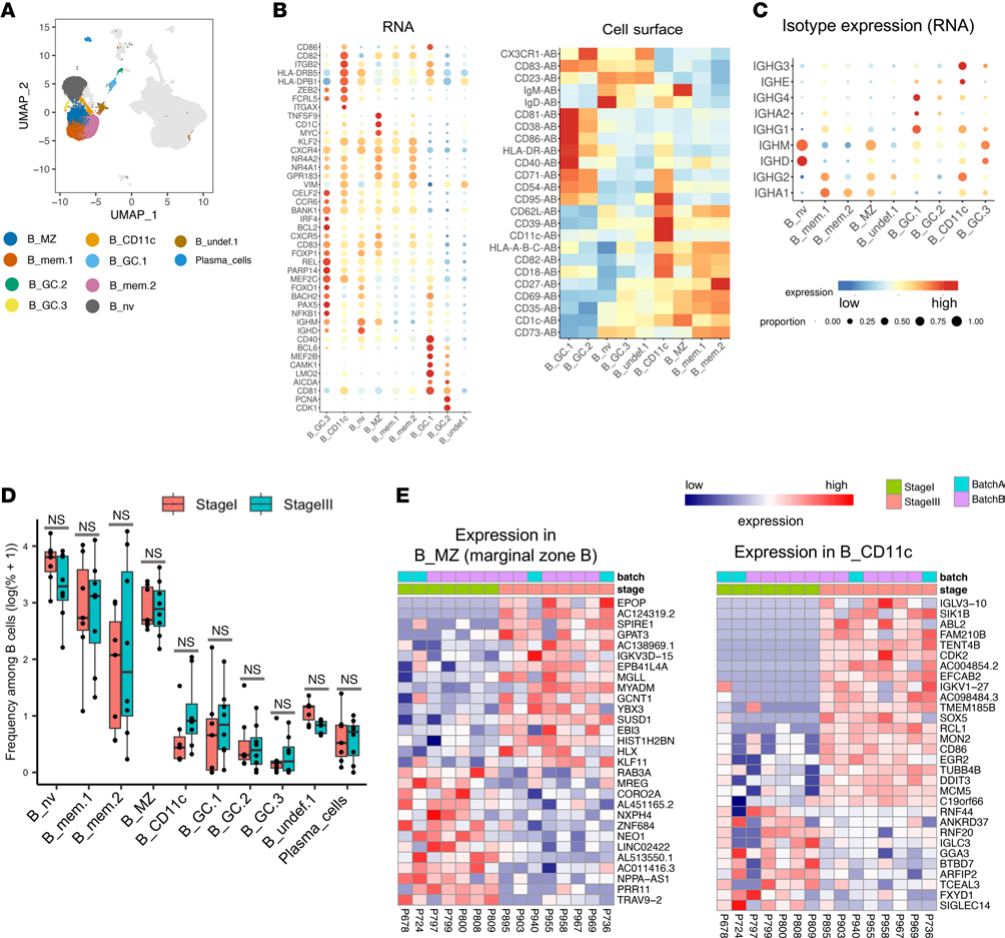
Characterization of B cell populations in melanoma SLN tissues. (**A**) UMAP plot with B cells highlighted. (**B**) Dot plots of RNA (left) and heatmap of cell surface (right) expression for selected genes upregulated in B cell clusters. (**C**) Dot plot showing expression of genes encoding antibody isotypes. (**D**) Box plot (median with interquartile range) showing the frequency of each B cell cluster among all B cells. (**E**) Heatmaps showing expression of all transcripts that are differentially expressed (*P* value < 0.01 and |log_2_ fold-change| > 0.8) between stage I and stage III SLN samples in marginal zone and CD11c^+^ B cells. Two-group comparisons were done by 2-tailed Student’s *t* tests.

We identified B cell populations known to reside in LNs, including cells associated with germinal centers, naive B cells, and memory B cells (Figure 4, A and B). The memory B cell cluster termed B_mem.2 expressed high levels of *NR4A1*, *NR4A2*, *GPR183*, *KLF2*, *CXCR4*, *CXCR5*, and CD27 akin to a B cell population recently described in rheumatoid arthritis synovium (55), whereas B_mem.1 showed high expression of *IGHG2* (Figure 4, B and C). RNA encoding IgA was detected in each of the 2 memory B cell populations (Figure 4C). A cluster of B cells termed B_undef.1 lacked distinctive RNA markers but exhibited high expression of ribosomal RNAs (Supplemental Table 7) and displayed high cell surface expression of CX3CR1 and CD23 (Figure 4B). While the function and identity of these cells are unclear, there was a trend toward decreased proportion of these CX3CR1^+^ B cells in MEL^+^ SLN tissues (Figure 4D). Also, a previous study identified a similar B cell cluster in human tonsil tissues (56).

Marginal zone B cells were identified by RNA and cell surface expression of CD1c and IgM (Figure 4, B and C) (57, 58). Marginal zone B cells from MEL^+^ SLNs showed upregulation of genes associated with B cell leukemia (*YBX3*) (59) and virus-induced transformation (*HLX*, *EBI3*) (60, 61) (Figure 4E), suggesting that SLN marginal zone B cell activation increases with tumor progression.

Germinal center B cells were identified as 3 clusters: B_GC.1, B_GC.2, and B_GC.3. Two of these clusters, B_GC.1 and B_GC.2, expressed AID (*AICDA*), which is critical for class-switch recombination and somatic hypermutation, as well as *CD81*, *LMO2*, *MEF2B*, *CAMK1*, and *BCL6*, whereas B_GC.3 showed high expression of *PAX5*, *MEF2C*, *FOXO1*, *FOXP1*, *IRF4*, *BANK1*, *REL*, *NFKB1*, *CELF2*, *CCR6*, *BCL2*, *CXCR5*, and *CD83* (Figure 4B); these genes have been described as prototypical germinal center B cell markers by others (56, 62–65). Several genes expressed by B_GC.3 have been associated with the germinal center dark zone (*FOXP1*, *FOXO1*, *CXCR4*) as well as precursor-memory and intermediate cells (*CCR6*, *CELF2*, *BANK1*, *CXCR4*) (Figure 4B). In contrast, the B_GC.1 and B_GC.2 cluster markers *LMO2* and *MEF2B* are associated with germinal center light zone B cells (56). B_GC.2 cells expressed a clear signature of proliferation (*CDK1*, *PCNA*), whereas B_GC.1 cells expressed genes involved in T cell activation (*CD40*, *CD86*) (Figure 4B). Consistent with low AID/*AICDA* expression in the B_GC.3 cluster, these cells harbored relatively low expression of immunoglobulin constant genes (Figure 4, B and C). Each of the 3 germinal center B cell types trended toward increase in proportion in MEL^+^ SLNs (Figure 4D).

A cluster of CD11c^+^ B cells was identified (Figure 4, A and B) in the SLN tissue, and the frequency of these cells trended toward increase in MEL^+^ SLNs (Figure 4D). CD11c^+^ B cells are mainly memory cells that, upon activation, can become antibody-secreting cells and are reportedly increased in frequency in multiple autoimmune and infectious disease contexts (66–68). CD11c^+^ B cells were identified by previously described cell surface (CD11c/*ITGAX*, CD95, CD18/*ITGB2*) and RNA (*FCRL5*, *ZEB2*, *ITGB2*/CD18, *ITGAX*/CD11c) markers (Figure 4B). In MEL^+^ SLNs, CD11c^+^ B cells showed displayed upregulation of *SOX5*, which is associated with CD11c^+^ B cell identity (66), as well as upregulation of the costimulatory molecule *CD86* (ligand for T cell CD28 and CTLA4) and the cyclin-dependent kinase 2 (Figure 4E). These data suggest a role for CD11c^+^ B cells in shaping T cell responses in the metastatic SLN tissues.

### Interpatient SLN heterogeneity occurs along an axis from naive to activated that is independent of disease stage.

**Figure 5:**
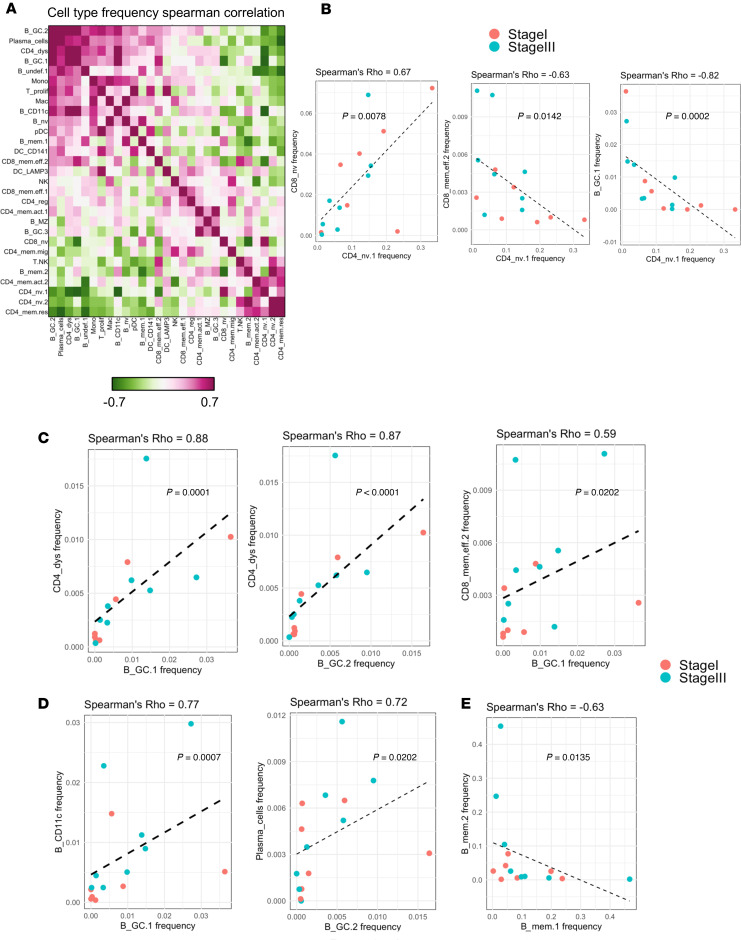
Melanoma SLN heterogeneity appears along an axis of naive to activated states. (**A**) Heatmap showing Spearman’s correlations between cell type frequencies computed from all SLN biopsies (*n* = 15). (**B**) Correlation between CD4^+^ naive (*x* axis) and CD8^+^ naive (left), CD8^+^ memory effector_2 (middle), and B_GC.1 (germinal center B cells; right). (**C**) Correlation between indicated germinal center B cell populations (*x* axis) and dysfunctional CD4^+^ T cells (left, middle) and CD8^+^ memory effector_2 cells (right). (**D**) Correlation between indicated germinal center B cell populations (*x* axis) and CD11c^+^ B cells (left) and plasma cells (right). (**E**) Correlation between 2 subtypes of memory B cells.

Single-cell approaches can reveal tumor subtypes with respect to cellular composition (69, 70). To determine whether there are SLN immune subtypes, we performed cell frequency correlation analysis among all patient samples (*n* = 15) (Figure 5A and Supplemental Table 8). Broadly, SLN subtypes could be described along an axis of naive and active; for example, naive CD4^+^ cells were positively correlated with naive CD8^+^ cells and negatively associated with both CD8^+^ effector memory cells and germinal center B cells (Figure 5B). Notably, unbiased identification of a naive → activated SLN subtype axis lends support to the notion that a specific LN memory signature portends improved survival among patients in The Cancer Genome Atlas dataset (71). High frequency of dysfunctional CD4^+^ and effector CD8^+^ T cells are likely features of an active SLN subtype, as these cells were positively correlated with germinal center B cells (Figure 5C). Similarly, CD11c^+^ B cells and plasma cells also positively correlated with germinal center B cells (Figure 5D). A negative correlation between B_mem1 and B_mem2 B cell subtypes (Figure 5E) suggests that the 2 memory B cell subsets represent distinct populations of antigen-experienced B cells; the extent to which this phenomenon can be explained by features of B cell receptor sequences is not clear.

### DC heterogeneity in melanoma SLNs.

**Figure 6:**
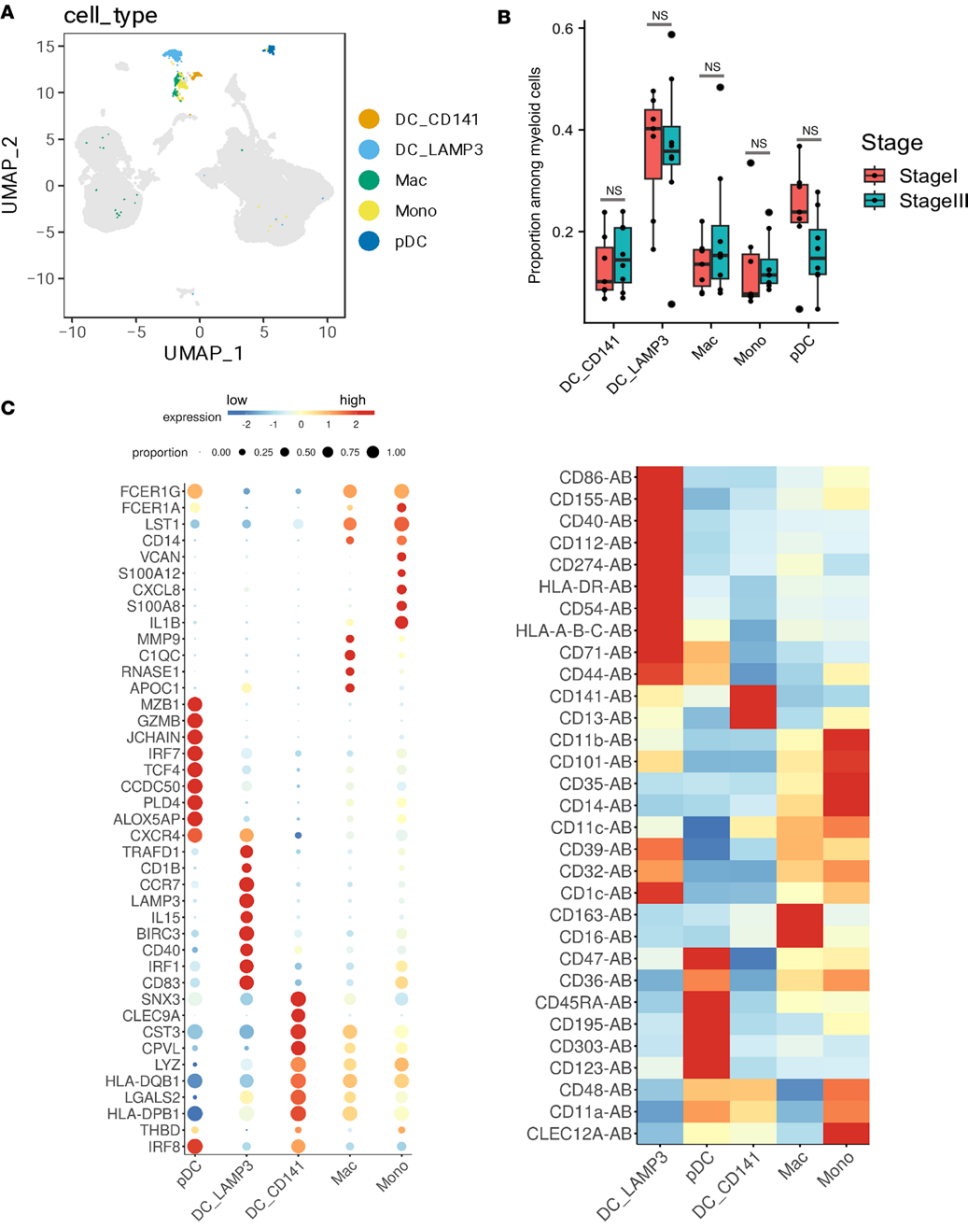
Differential transcript and protein marker expression of myeloid cell populations in melanoma SLN tissues. (**A**) UMAP plot with myeloid cells highlighted. (**B**) Box plot of frequencies of myeloid cell types (among all myeloid cells) in SLN biopsies. Box plot edges indicate interquartile range. (**C**) Dot plots of RNA (left) and heatmap of cell surface (right) expression for selected genes upregulated in myeloid cell clusters. Two-group comparisons were done by 2-tailed Student’s *t* tests.

Within the myeloid cell compartment, we identified 5 subsets of cells that included 3 distinct subsets of DCs and small clusters of macrophages and monocytes (Figure 1, C and D, and Figure 6, A and B). Two of the 3 distinct DC clusters mapped to the well-established conventional CD141^+^ DC1s (cDC1s) and pDCs while the third cluster corresponded to a CCR7^+^LAMP3^+^ DC subset.

The genes specifically expressed by pDCs, but not by cDCs, were associated with the known biological properties of pDCs. These genes include those involved in pathogen sensing and induction of type I IFNs (*IRF7*, *TLR7*, *IRF8*), as well as the pDC master regulator transcription factor (*TCF4*) (Figure 6C). At the protein level, pDCs in SLNs expressed the prototypical pDC markers CD123 and CD303 (Figure 6C). Interestingly, pDCs also expressed the gene that encodes for serine protease Granzyme B (Figure 6C). Previous work has shown that pDCs expressing Granzyme B exhibit a marked ability to inhibit the proliferation of T cells (72). This inhibition is reliant on Granzyme B but does not depend on perforin, akin to the suppressive activity of Tregs. SLN pDCs expressed phospholipase D4 (*PLD4*), a glycosylated type II transmembrane protein located in endolysosomes (Figure 6C). PLD4 is a 5′ exonuclease that degrades single-stranded DNA and thereby regulates exogenous TLR8/9-mediated type I IFN response in pDCs (73). pDCs expressed *CXCR4*, a 7-transmembrane-spanning G protein–coupled receptor (Figure 6C) (74). The enhanced secretion of its chemokine ligand, CXCL12, by tumor cells correlates positively with the LN metastasis of these tumor cells (75). Other unique genes expressed by SLN pDCs include *CCDC50*, an autophagy receptor that negatively regulates STING-directed type I IFN signaling activity (76); *MZB1*, an endoplasmic reticulum–residing protein that enables pDCs to secrete high amounts of IFN-α by mitigating ER stress via increased unfolded protein response, a process that is reminiscent of plasma cell differentiation (77); and *ALOX5AP*, arachidonate 5-lipoxygenase activating protein, which is important for leukotriene biosynthesis (Figure 6C).

Our findings revealed distinct variations in the expression of HLA molecules across various DC subsets in the SLN tissue, indicating subset-specific differences in antigen presentation. Specifically, the CD141^+^ cDC1 subset exhibited high expression levels of *HLA-DQ* and *HLA-DP* when compared with those in pDCs (Figure 6C).

Our data also revealed a cluster of DCs with a transcriptional profile that is identical to DC3, a subset of mature DCs enriched in immunoregulatory molecules (mregDCs) (Figure 6A). In many tumor models, mregDCs are considered a DC subset that inhibits antitumor immunity. In agreement with previous studies (78, 79), mregDCs in SLNs expressed *LAMP3* along with *CCR7*, CD274 (PD-L1), and immune costimulatory maturation markers CD40, CD83, and CD86 and lacked expression of key cDC1, cDC2, and pDC markers (Figure 6C). mregDCs in SLNs also expressed high levels of CD1c, immunosuppressive CD39, HLA-DR, CD112, CD155, and CD54 surface markers (Figure 6C). LAMP3^+^ mregDCs have been shown to be commonly present in the microenvironment of different tumors, arising from cDCs through maturation in the tumor microenvironment; to traffic to LNs; and to contain an immunoregulatory transcriptional module that can suppress T cell functions and, thus, can promote tumor metastasis.

## Discussion

The first step in treating patients with clinically node-negative melanoma involves wide local excision of the primary tumor along with SLN biopsy. SLN biopsy is critical for staging and reducing the risk of regional nodal melanoma recurrence. Moreover, SLN tissue can be an active immune site with potential to prime locoregional and systemic antitumor responses with immunological memory. Surgically removing the SLN tissue before initiating immunotherapy might result in loss of immune subsets that are capable of mounting a systemic immune response to eliminate melanoma. There is growing consensus that neoadjuvant immunotherapy has a place in melanoma management, though precise therapeutic regimens are under investigation and may require tailoring to patient-specific features, such as tumor mutation profile, tumor microenvironment, or LN immune profile. Immunoprofiling can help unravel the nature of the immune potential of the SLNs. Here, using CITE-Seq, we provide a line of evidence that SLN is an actionable microenvironment. Specifically, we describe the transcriptomic features of a multitude of T, B, and myeloid cell subtypes in SLNs of stage I and stage III patients.

In the T cell compartment, we identified CD8^+^ effector memory T cell (CD8_EM2) and T/NK populations that were more frequent in stage III MEL^+^ SLN tissues, suggesting an active immune response. A more abundant CD8^+^ effector memory subtype (CD8_EM1) showed evidence of transcriptomic alteration between stages I and III, with notable enrichment of transcripts associated with T cell exhaustion/suppression (e.g., TOX2, NR4A1) in stage III samples. Similarly, a migration-associated CD4^+^ T subset also showed upregulation of genes associated with T cell exhaustion (e.g., PDCD1/PD-1, CTLA4, TOX2), collectively indicating that tumor progression in stage III patients correlates with features of T cell exhaustion. In both stage I and III samples, CD4^+^ T cell subsets, including Tregs, expressed specific combinations of checkpoint genes that partially overlapped with those observed in the dysfunctional CD4^+^ T pool. Interestingly, our data also identified a cluster of CD4^+^PD-1^+^TCF-1^+^ progenitor exhausted cells that retain polyfunctionality, persist long-term, and exhibit superior ability to control tumor growth. Recent studies have provided important evidence that progenitor exhausted T cells are more responsive to anti–PD-1 therapy than terminally exhausted T cells (50–53). Furthermore, in patients with melanoma, a higher proportion of progenitor exhausted cells correlates with an extended duration of response to ICB therapy (50). Consequently, strategies aimed at expanding the population of progenitor exhausted CD4^+^ T cells in the SLN tissues may prove crucial to increasing responsiveness to ICB therapy and controlling melanoma progression.

Our identification of CD4^+^ T cells with cytolytic features is in agreement with a previous study that identified such cells in melanoma primary tumors and tumor-infiltrated draining LNs (54); we extend this finding by identification of CD4^+^GZMA^+^ T cells in SLNs, suggesting that these cells can play a role in antitumor immunity early in the course of disease. In MEL^+^ SLNs, the identified pool of cytotoxic CD4^+^ and CD8^+^ T cells could play a bystander role and be irrelevant to eradicating the melanoma cells that reside in the SLN tissue. Previous studies (80, 81) have presented evidence supporting the presence of such bystander cells within human tumors. A second possibility is that the cytotoxic T cell pool is tumor reactive but may display a poor melanoma antigen–specific priming in terms of both magnitude and quality of the response.

Our data support the notion that the CXCR3 chemokine system holds functional importance for tissue-resident CD8^+^ T cells and may influence the anti–PD-1–mediated response of CD8^+^ T cells residing within the SLNs. Furthermore, we found an increase in the levels of circulating host-derived CXCL10 plasma levels in stage III patients, which may be a positive predictor of a therapeutic response to anti–PD-1 blockade in these patients. Future investigations will be needed to decipher the intricate interactions among various T cell populations within a larger framework that encompasses interactions with other cells in SLNs as well as with those present in tumor cells. Understanding these interactions will shed light on their potential immunomodulatory roles in the context of melanoma progression as well as clinical responses to neoadjuvant immunotherapies.

Discerning disease subtypes and corresponding treatment regimens is the challenge of precision medicine. Here, we used cell type proportion information and determined that SLN subtypes are stratified along a naive → activated axis; SLN tissues with a high proportion of naive T cells had a lower proportion of effector CD8^+^ T and germinal center B cells and vice versa. It is possible that patients with a “high activated” signature score are undergoing a more robust melanoma antigen–specific adaptive immune response and may be most responsive to intervention with ICB therapy.

In the context of antitumor immunity, an active B cell response, including tertiary lymphoid structures (82), has been associated with positive immunotherapy responses. Relatively high proportions of CD11c^+^ B cells in both activated subtypes and melanoma-bearing SLN samples may suggest a role in antitumor immunity; however, analysis of these cells coupled with adaptive immune receptor sequencing in larger, longitudinal studies will likely be needed to address the role of CD11c^+^ B cells in the SLN tissues.

Among the different myeloid cell subsets, DCs play an important role in orchestrating adaptive immune responses in the LNs. We present a comprehensive comparison of DC subsets in the melanoma SLN tissues. Our CITE-Seq data resolved DC profiles into 3 subsets, cDC1, pDCs, and mregDCs (78, 83, 84). mregDCs, which have recently been described in tumors, are a subset of mature DCs with different names, resulting in their classification as LAMP3^+^ DCs, CCR7^+^ DCs, or BATF3^+^ DCs; however, all describe the same DC subset. Increased mature LAMP^+^ DCs in SLNs have previously been found to correlate with increased overall patient survival (85). With the expressed chemokine receptor CCR7, mregDCs can traffic the tumor antigens to the SLNs and prime antitumor T cell responses. In our study, mregDCs were the most prevalent myeloid subset in the SLN tissues whereas monocytes and macrophages were the least prevalent. Interestingly, mregDCs in SLN tissues expressed high levels of PD-L1 as well as immunosuppressive CD39 molecules, both of which can limit their antitumor responses. As such, therapeutic strategies that inhibit PD-1/PD-L1 while simultaneously licensing T cell–priming activity of DCs can be a powerful approach to unleash immunity against melanoma cells residing in SLNs. Among other DC subsets, our data show that CD141^+^ cDC1s that can crosspresent antigens to CD8^+^ T cells were strongly reduced in SLNs (5), while cDC2s that activate CD4^+^ T cells were not present in SLN tissues. It is plausible that the migration of cDC2s or their precursors might be impeded (86, 87). Additionally, tumor-derived factors within the local environment could directly obstruct the maturation or survival of DCs within the SLNs (88, 89). pDCs play a crucial role in regulating antitumor immune responses via production of a high amount of type I IFN and activation of TLR7 and -9 signaling pathways; however, melanoma cells in the SLN tissues can subvert the antitumor function of pDCs. Increased infiltration of pDCs into melanoma tumors and SLNs has been associated with poor prognosis and increased early relapse (90). Primary melanoma-infiltrating pDCs do not migrate to LNs via lymphatic vessels, and accordingly, we and others have observed a reduction in the number of pDCs in metastatic MEL^+^ SLNs. These observations lead to a hypothesis that a therapeutic reversion of tolerogenic functions of pDCs could pave the way for inducing antimelanoma responses in SLNs.

Taken together, the CITE-Seq dataset that we describe here provides a valuable resource to firmly establish the heterogeneity and functional organization of immune subsets present in the melanoma SLN tissues and to decipher the contribution of each subset to the initiation of antitumor immune responses in patients with stage I and stage III melanoma.

### Limitations of the study.

There were very few differences in cell type proportions, and these differences did not reach statistical significance, primarily because of the smaller sample size in this study (7 MEL^–^ SLNs and 8 MEL^+^ SLNs; 15 samples) compared with our previous study (5), which included 108 samples. However, the characteristics of different immune cell subsets identified from the CITE-Seq data are very much in alignment with that obtained from our earlier study (5). Overall, there was an expansion of effector memory T cells in melanoma-bearing SLNs. Furthermore, both of our studies identified a presence of an immunotolerant microenvironment in MEL^+^ SLNs characterized by enrichment of T cell subsets transitioning from an activated state to a more terminally differentiated, exhausted phenotype; NK cells expressing markers that suppress their cytotoxic activity; and lower prevalence of myeloid cells. Additionally, we did not observe any statistically significant differences in the proportions of B cells between MEL^+^ SLN and MEL^–^ SLN samples, which is in agreement with our previously published study (5). Importantly, our CITE-Seq study provides higher resolution of functional phenotypes of these immune cell types based on gene expression. Our previous study had identified a higher number of γδ T cells in MEL^+^ SLNs (5). However, in our current study, the clustering approach used in the transcriptome analysis did not yield a distinct γδ T cell cluster. One possibility is that γδ T cells could appear as part of the NK cell cluster. This could be due to an overlap of transcriptomic signatures between γδ T cells and NK cells as previously reported (91).

A longitudinal study that evaluates the recorded changes in immune signatures with disease progression would be invaluable for validating immune cell changes as prognostic markers of disease progression and predictors of immunotherapeutic outcomes. The collection of clinical follow-up data from patients enrolled in this study is ongoing (Supplemental Table 11), and data will be analyzed once sufficient data are available. Future studies will also investigate changes in immune cells in the context of disease progression using a mouse model of LN metastases to provide conclusive evidence supporting the use of these immune signatures as therapeutic targets.

## Methods

### Sex as a biological variable

SLN biopsies were collected from both male and female patients (Supplemental Table 1), as melanoma affects both men and women.

### Patient samples (SLN)

Human SLN tissue samples were obtained from patients with cutaneous melanoma who underwent SLN biopsy at the University of Louisville. All patients underwent SLN biopsy using a combination of radioactive tracer and isosulfan blue dye. A random portion of the most radioactive SLN (2 mm in greatest dimension or one-quarter of the SLN, whichever was smaller) from each patient was obtained fresh at the time of surgery. All patients with an SLN biopsy were either positive (pathologic stage III) or negative (pathologic stage I/II, referred to as stage I) for metastatic melanoma by pathological analysis of serial sections by H&E staining and immunohistochemical staining for MART-1 and SOX-10. Deidentified clinical information for the patients included in this study is provided in Supplemental Table 11. A portion of the SLN was enzymatically digested and cryopreserved in cryovials. Cryovials were then transferred to −150°C for storage. Details of SLN tissue processing and cryopreservation are provided in Supplemental Methods.

### Patient cohorts and study design

The collected SLN samples were divided into 2 cohorts. Please see Scheme I and related section in Supplemental Methods for details of the cohorts. Samples in cohort I (7 stage I and 8 stage III) were exclusively used for CITE-Seq analysis. Samples in cohort II (6 stage I and 6 stage III) were used for flow cytometry. In addition, serum samples were collected from 34 patients (cohort III; 18 stage I, 16 stage III) and 3 healthy donors and used exclusively for Bio-Plex analysis.

### CITE-Seq

Single-cell suspensions from SLN tissues obtained from stage I and stage III patients (cohort I) were washed twice and individually stained with CITE-Seq antibodies that were purchased from the BioLegend TOTALseq catalog (catalog 399905). Libraries were constructed using Chromium Single Cell 5′ Reagent Kits from 10x Genomics following the manufacturer’s user guide.

#### Gel Beads-in-emulsion generation and barcoding.

Cells were mixed with room temperature master mix and loaded onto Chromium Chip K (10x Genomics). A total of 16,500 cells per sample were loaded for a targeted recovery of 10,000 cells. After being loaded with gel beads and partitioning oil, the chip was run on the Chromium Controller using Chip K program. A total of 100 μL of Gel Beads-in-emulsion from each sample was recovered, then incubated for the room temperature reaction using a thermal cycler at 53°C for 45 minutes, then 85°C for 5 minutes followed by a 4°C hold.

#### cDNA amplification and cleanup.

Purified cDNA was amplified with a 13-cycle PCR program. Amplified cDNA was then cleaned up using a 0.6× SPRIselect magnetic bead (Beckman Coulter B23318) size selection. A total of 80 μL of the supernatant was further subjected to a 2.0× SPRIselect cleanup for cell surface protein library construction.

#### 5′ Gene expression library construction.

A total of 50 ng of amplified cDNA underwent enzymatic fragmentation, end repair, and A-tailing. The resulting product was cleaned up with a 0.6×–0.8×, double-sided size selection using SPRIselect magnetic beads. Then 50 μL of purified product was transferred to a new tube. For adaptor ligation, 50 μL of freshly prepared Adaptor Ligation Mix was added to each sample and incubated at 20°C for 15 minutes followed by a 4°C hold. Illumina R2 sequence was added during this step. Following ligation, the samples were purified using a 0.8× SPRIselect magnetic bead cleanup. Then 30 μL of purified product was transferred to a new tube. Next, samples were indexed with individual i5 and i7 dual indexes (Dual Index Kit TT Set A) through a 14-cycle PCR. P5 and P7 were also added during this PCR. Indexed libraries were purified with a 0.6×–0.8×, double-sided size selection using SPRIselect magnetic beads.

#### Cell surface protein receptor mapping library construction.

A total of 5 μL sample from the transferred supernatant cleanup was mixed with PCR mix, and individual i5 and i7 dual indexes (Dual Index Kit TN Set A), and amplified for 7 cycles. P5, P7, and Nextera Read 2 were also added during this PCR. Indexed libraries were purified with a 1.2× SPRIselect magnetic bead cleanup.

#### Sample and barcode information.

We barcoded samples with Dual Index Kit TT Set A and Dual Index Kit TN Set A. We analyzed 1:10 diluted libraries on a Bioanalyzer DNA High Sensitivity Chip (Agilent Technologies, catalog 5067-4626).

#### Normalizing and pooling libraries.

The same amount of same type of libraries were pooled based on the molar concentration from Bioanalyzer.

#### Library denaturing and NextSeq 500 sequencing.

Equal amounts of the same type of libraries were repooled for NextSeq runs. The 5′ gene expression and cell surface protein libraries were pooled in a ratio of 1:4:1. Library pool and PhiX were denatured and diluted using the standard normalization method following manufacturer’s directions. Sequencing type was paired-end, dual indexing. Sequencing reads were read 1: 26 cycles, i7 index: 10 cycles, i5 index: 10 cycles, read 2: 122 cycles. Sequencing was performed on the Illumina NextSeq 500 using the NextSeq 500/550 150 cycle High Output Kit v2.5 (20024907).

### CITE-Seq analysis

The Cell Ranger (v3.1.0) software provided by 10x Genomics was used to generate barcodes.tsv, features.tsv, and matrix.mtx files for each of 2 runs (10x Genomics). The count matrix from each 10x Genomics run was loaded into Seurat (v4.0) using the Read10x function, and sample-level metadata were added to Seurat objects using the AddMetaData function.

Count matrices were filtered using the following R code: subset(<seurat_object>, subset = nCount_RNA >= 1000 & nFeature_RNA > 500 & nFeature_RNA < 3000 & percent.mt < 10), where “percent.mt” corresponds to percentage of counts (unique RNA molecules) mapped to a mitochondrial transcript. The 2 count matrices were normalized using the NormalizeData command; variable features from the RNA assay were identified using the FindVariableFeatures command with the options ‘selection.method = “vst,” nfeatures = 5000’; and the 2 count matrices were integrated using the RNA assays with the following commands: SelectIntegrationFeatures with options ‘nfeatures = 5000, fvf.nfeatures = 5000’; FindIntegrationAnchors with option ‘reduction = “rpca,”’ and IntegrateData with default options. The command “ScaleData” was run on the integrated object with the option ‘vars.to.regress = “percent.mt.”’ Then clustering was performed using the commands “RunPCA,” “RunUMAP,” “FindNeighbors,” and “FindClusters” with option ‘resolution = 0.9.’ RNA expression data with cluster assignments for each cell and associated sample-level metadata are available at https://eengelbrecht.shinyapps.io/Melanoma_SLN_RNA/, and cell surface protein expression data are available at https://eengelbrecht.shinyapps.io/Melanoma_SLN_ADT/, wherein cell types are codified in the “cell_type” drop-down option. For each cluster, average RNA and cell surface (ADT assay) expression was computed using the “AverageExpression” command from Seurat (Figure 1, C and D). Expression heatmaps were generated using the dittoSeq package (92). For a given cluster, expression plots include cells from all donors unless indicated otherwise. Cell type correlations (Figure 1F) were computed using the “clustify” command of the clustifyr package (93) with default parameters. The “wilcoxauc” command from the presto package (https://github.com/immunogenomics/presto/; commit ID 7636b3d) was used for identification of cluster markers (Supplemental Tables 2–5 and 7). Expression comparisons within cell type groupings from stage I (SLN^–^) and stage III (SLN^+^) were computed using DESeq2 (94) on pseudobulk data generated using the following R code: Seurat:AggregateExpression(<seurat_object>, group.by = c(“cell_type”, “Sample_ID”, “Batch”), assays = ‘RNA’, slot = “counts”, return.seurat = FALSE). Heatmaps of gene expression were generated using pheatmap (95). Spearman’s correlations of cell type frequencies between patient samples (Figure 5 and Supplemental Table 8) were determined using the cor() function in R.

### Multiplex Bio-Plex cytokine and chemokine assay

Levels of cytokines and chemokines in serum samples were measured using the Bio-Plex Pro Human Cytokine Screening Panel 48-Plex assay. Serum samples (cohort III) were collected from healthy donors (*n* = 3), patients with stage I melanoma (*n* = 18), and patients with stage III melanoma (*n* = 16) and stored at –150°C. Samples were thawed at room temperature on the day of the assay, and analytes were measured per assay manufacturer’s instructions. Cytokine and chemokine analytes measured are provided in Supplemental Methods. Assay controls included Bio-Plex kit standards and multiplexed external cytokine standards. For all measurements, fluorescent bead–based instrument Bio-Plex 200 (Bio-Rad) was used according to the manufacturer’s protocol.

### Flow cytometry

Single-cell suspensions from stage I and stage III (cohort II) were washed twice and resuspended in FACS buffer (2% FBS + PBS) and stained with multicolor antibody cocktails according to the manufacturer’s recommendations. Stained cells were analyzed with a FACSCanto II (BD Biosciences), and data were analyzed using FlowJo v10 (BD Biosciences). FACS dot plots are represented with log-scale axes. Histograms are represented on a log-scale *x* axis and a linear *y* axis. Details on antibody panels are provided in Supplemental Methods.

### Statistics

Flow cytometry and Luminex data were analyzed using GraphPad Prism 8.0 software. Two-group comparisons between control and test samples (groups compared are indicated in the respective figures) were done by 2-tailed Student’s *t* tests. Cell proportions and the *P* values are provided in Supplemental Table 10. *P* < 0.05 was considered statistically significant. Multiple data comparisons were derived by 1-way ANOVA followed by Tukey’s post hoc test.

### Study approval

Human SLN tissue samples were collected after informed written consent was obtained from all patients on a protocol that was approved by the University of Louisville Institutional Review Board (number: 08.0491). These studies were conducted in accordance with US Common Rule.

### Data availability

Cell-level RNA expression and cell surface protein expression data, in addition to cell-level metadata including cluster designations, are publicly available through Zenodo at https://doi.org/10.5281/zenodo.14775656 Gene expression data can be interactively explored at https://eengelbrecht.shinyapps.io/Melanoma_SLN_RNA/ for RNA data and at https://eengelbrecht.shinyapps.io/Melanoma_SLN_ADT/ for cell surface protein expression data, with cell types annotated for each cell in the “cell_type” drop-down option. The data generated in this study are available within the article and its supplemental files where indicated and in the Supporting Data Values file.

## Author contributions

KY and CW conceived the experiments. EE, ECR, and J Chariker performed the CITE-Seq data analysis. BFS, LC, OSS, PH, and SJW conducted the experiments. AS, J Chesney, and KM provided reagents and resources. EE, KY, and CTW analyzed the results. KY and EE wrote the manuscript, and CTW reviewed and edited the manuscript.

## Supplementary Material

Supplemental data

Supplemental tables 1-11

Supporting data values

## Version 1 03/06/2025

In-Press Preview

## Version 2 04/08/2025

Electronic publication
