# Supplementary material for: Single-cell transcriptomics of melanoma sentinel lymph nodes identifies immune cell signatures associated with metastasis
Source: JCI Insight. 2025 Mar 6;10(7):e183080. doi: 10.1172/jci.insight.183080 (PMC11981627; doi:10.1172/jci.insight.183080)
Supplement: Supplemental data [file jciinsight-10-183080-s064.pdf]

Supplemental Figure 1

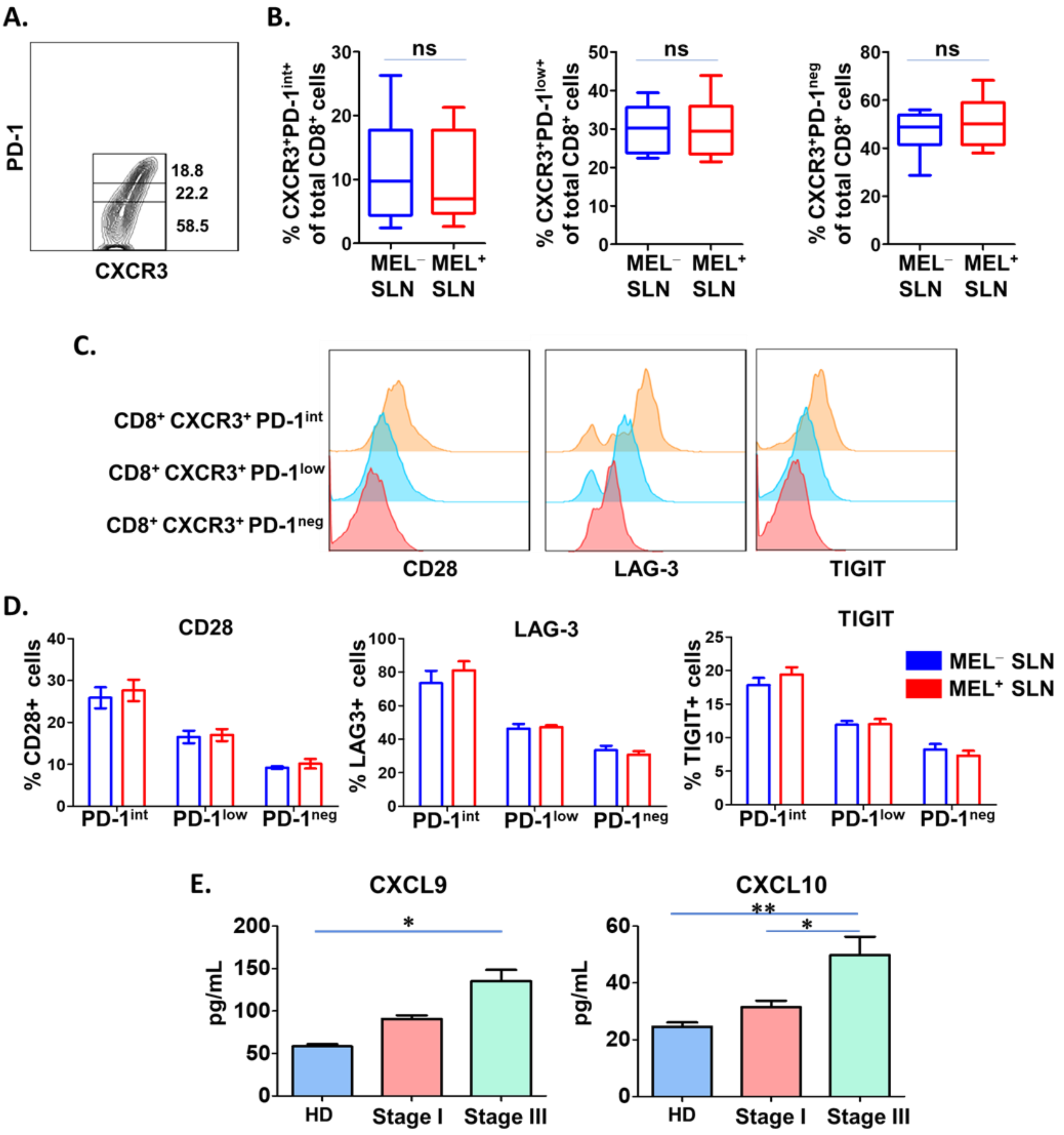

Supplemental Figure 1. A–D. **Identification of three subsets of CD8⁺ T cells within SLN based on the cell surface expression of CXCR3 and PD-1. A, B.** SLN samples from stage I (MEL⁻ SLN; n = 6) and stage III (MEL⁺ SLN, n = 6) melanoma patients were analyzed for the expression of immune cell markers by flow cytometry. **A, B.** Representative contour plot (**A**) and bar graphs (**B**) show the identification of three subsets of CD8⁺ T cells within the MEL⁻ and MEL⁺ SLN tissue based on CXCR3 and PD-1 expression. **C, D.** Histograms (**C**) and bar graphs (**D**) showing the expression of CD28, LAG-3, and TIGIT on the three subsets of CD8⁺ T cells – CD8⁺CXCR3⁺PD-1ⁱⁿᵗ, CD8⁺CXCR3⁺PD-1ˡᵒʷ, and CD8⁺CXCR3⁺PD-1ⁿᵉᵍ within the SLN tissues. **E. Stage III melanoma patients have elevated circulating levels of CXCL9 and CXCL10 chemokines.** Levels of CXCL9 and CXCL10 in serum samples collected from healthy donors (n = 3), stage I melanoma patients (n = 18), and stage III melanoma patients (n = 16). One of the serum samples was excluded from the analysis due to the presence of a contaminant in the sample (see **Table S9**). Data, mean ± SEM, *P* values: *, p≤0.05; **, p≤0.005.

Supplemental Methods

Patient Cohorts and Study Design. For this study, one SLN sample was obtained from each of 27 patients (13 stage I and 14 stage III). These samples were divided into two cohorts (Cohort I and II). In addition, serum samples were collected from 34 patients (Cohort III; 18 stage I and 16 stage III) (Please see **Supplemental Scheme I** and **Table 1** (1a, 1b, and 1c; see below) for details of the Cohorts.

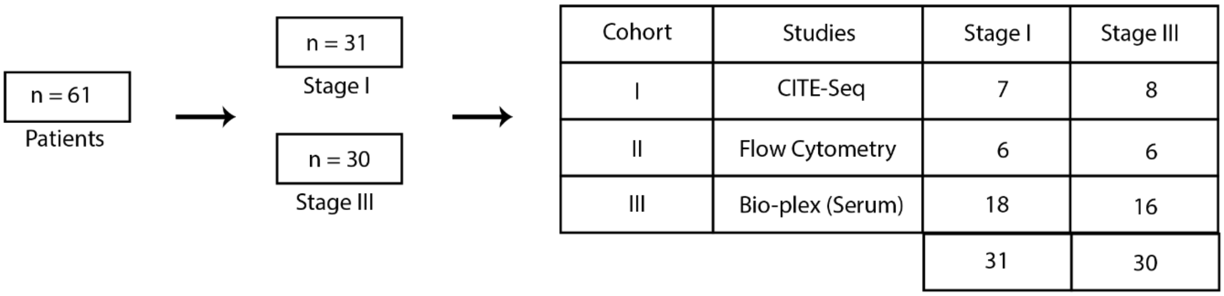

Supplemental Scheme 1: Details of SLN and serum sample Cohorts.

Table 1: Cohort Details

Table 1a: Cohort I (CITE-Seq)					
#	Specimen Code*	Stage	Gender	Age	Experiment
1	678	I	M	50	CITE-Seq
2	724	I	F	50	CITE-Seq
3	797	I	F	29	CITE-Seq
4	799	I	F	61	CITE-Seq
5	800	I	F	62	CITE-Seq
6	808	I	M	70	CITE-Seq
7	809	I	M	61	CITE-Seq
8	736	III	F	40	CITE-Seq
9	895	III	F	67	CITE-Seq
10	903	III	M	41	CITE-Seq
11	940	III	F	39	CITE-Seq
12	955	III	M	75	CITE-Seq
13	958	III	F	39	CITE-Seq
14	967	III	F	47	CITE-Seq
15	969	III	M	76	CITE-Seq

***Samples attributed to the missing ID numbers were not used in the study for one of the following reasons: a) not collected, b) low viability, or c) low cell number.**

Table 1b: Cohort II (Flow Cytometry)					
#	Specimen Code*	Stage	Gender	Age	Experiment
1	634	I	M	58	Flow Cytometry
2	635	I	F	52	Flow Cytometry
3	636	I	M	55	Flow Cytometry
4	704	I	M	90	Flow Cytometry
5	705	I	F	52	Flow Cytometry
6	706	I/II	M	53	Flow Cytometry
7	537	III	M	42	Flow Cytometry

8	549	III	F	31	Flow Cytometry
9	655	III	F	64	Flow Cytometry
10	721	III	F	88	Flow Cytometry
11	729	III	F	30	Flow Cytometry
12	734	III	M	63	Flow Cytometry

Table 1c: Cohort III (Serum-Bio-plex assay)

#	Specimen Code*	Stage	Gender	Age	Sample	Experiment
1	596	I	M	84	Serum	Bio-plex
2	598	I	F	53	Serum	Bio-plex
3	603	I	F	67	Serum	Bio-plex
4	605	I	M	39	Serum	Bio-plex
5	608	I	M	57	Serum	Bio-plex
6	611	I	M	76	Serum	Bio-plex
7	612	I	M	44	Serum	Bio-plex
8	620	I	F	38	Serum	Bio-plex
9	627	I	F	72	Serum	Bio-plex
10	628	I	F	28	Serum	Bio-plex
11	636	I	M	55	Serum	Bio-plex
12	641	I	M	86	Serum	Bio-plex
13	644	I	M	68	Serum	Bio-plex
14	650	I	M	53	Serum	Bio-plex
15	676	I	F	49	Serum	Bio-plex
16	677	I	M	49	Serum	Bio-plex
17	680	I	F	43	Serum	Bio-plex
18	687	I	M	67	Serum	Bio-plex
19	601	III	M	52	Serum	Bio-plex
20	617	III	M	70	Serum	Bio-plex
21	618	III	M	62	Serum	Bio-plex
22	633	III	F	77	Serum	Bio-plex
23	639	III	F	66	Serum	Bio-plex
24	643	III	F	79	Serum	Bio-plex
25	651	III	M	81	Serum	Bio-plex
26	657	III	M	36	Serum	Bio-plex
27	660	III	M	39	Serum	Bio-plex
28	683	III	M	47	Serum	Bio-plex
29	684	III	M	49	Serum	Bio-plex
30	694	III	M	62	Serum	Bio-plex
31	695	III	F	39	Serum	Bio-plex
32	700	III	M	55	Serum	Bio-plex
33	708	III	M	67	Serum	Bio-plex
34	721	III	F	88	Serum	Bio-plex

SLN Digestion – A portion of the SLN was enzymatically digested in RPMI 1640 medium (Corning Life Sciences, Corning, NY) containing 5% FBS (Thermo Fisher Scientific, Waltham, MA), 1% L-glutamine (Corning Life Sciences), 1% penicillin/streptomycin (Corning Life Sciences), 0.1% DNase (Thermo Fisher) and collagenase type IV (1 mg/mL; Sigma-Aldrich, St. Louis, MO) for 30 minutes at 37 °C. The single cell suspensions were then filtered through a 70 μm cell strainer, washed, lysed in RBC buffer (BioLegend, San Diego, CA), re-filtered through a 70 μm

cell strainer and cryopreserved. For cryopreservation, SLN cells were resuspended in cooled (4 °C) freezing media (90% FBS and 10% DMSO) and transferred into cryovials. Once aliquoted, cryovials were transferred into a freezing container (Nalgene, Rochester, NY), and stored at −80 °C for 24 hours. Cryovials were then transferred to −150 °C for storage.

Flow Cytometry – For fluorescence-activated cell sorting (FACS) analysis of the frequency and phenotype of SLN tissues, cryopreserved patient SLN samples were thawed. Cells were then washed twice and resuspended in FACS buffer [2% FBS + PBS] and stained with multi-color antibody (Ab) panels (related to Figure 3). The antibody panels used for staining are shown in the table below:

Table 2: Flow Cytometry Antibodies:

Antibody	Color	Supplier	Clone	Catalog #
CD4	FITC	Biolegend	OKT4	317408
CD8	APC/Cy7	Biolegend	RPA-T8	301016
CD279 (PD-1)	APC	Biolegend	EH12.2H7	329908
CXCR3	PerCPCy5.5	Biolegend	G025H7	353714
CD28	PE/Cy7	Biolegend	CD28.2	302926
LAG3	FITC	Biolegend	11C3C65	369308
TIGIT	PE/Cy7	Biolegend	VSTM3	372714

Table 3: Bioplex 40-plex Human Analytes:

Number	Bioplex Analytes
1	Hu IL-1b (39)
2	Hu IL-2 (38)
3	Hu IL-4 (52)
4	Hu IL-6 (19)
5	Hu IL-8 (54)
6	Hu IL-10 (56)
7	Hu Eotaxin (43)
8	Hu GM-CSF (34)
9	Hu IFN-γ (21)

10	Hu IP-10 (CXCL10) (48)
11	Hu MCP-1(MCAF) (53)
12	Hu MIP-1a (55)
13	Hu TNF-α (36)
14	Hu 6Ckine/CCL21 (12)
15	Hu BCA-1/CXCL13 (74)
16	Hu CTACK/CCL27 (72)
17	Hu ENA-78/CXCL5 (73)
18	Hu Eotaxin-2/CCL24 (30)
19	Hu Eotaxin-3/CCL26 (65)
20	Hu Fractalkine/CXCL1 (77)
21	Hu GCP-2/CXCL6 (15)
22	Hu GRO-a/CXCL1 (61)
23	Hu GRO-b/CXCL2 (78)
24	Hu I-309/CCL1 (20)
25	Hu IL-16 (27)
26	Hu I-TAC/CXCL11 (25)
27	Hu MCP-2/CCL8 (57)
28	Hu MCP-3/CCL7 (26)
29	Hu MCP-4/CCL13 (28)
30	Hu MDC/CCL22 (29)
31	Hu MIF (35)
32	Hu MIG/CXCL9 (14)
33	Hu MIP-1d/CCL15 (66)
34	Hu MIP-3a/CCL20 (62)
35	Hu MIP-3b/CCL19 (76)
36	Hu MPIF-1/CCL23 (37)
37	Hu SCYB16/CXCL16 (64)
38	Hu SDF1a+b/CXCL12 (22)
39	Hu TARC/CCL17 (67)
40	Hu TECK/CCL25 (46)
